# Fragility fracture identifies patients at imminent risk for subsequent fracture: real-world retrospective database study in Ontario, Canada

**DOI:** 10.1186/s12891-021-04051-9

**Published:** 2021-02-26

**Authors:** Jonathan D. Adachi, Jacques P. Brown, Emil Schemitsch, Jean-Eric Tarride, Vivien Brown, Alan D. Bell, Maureen Reiner, Millicent Packalen, Ponda Motsepe-Ditshego, Natasha Burke, Lubomira Slatkovska

**Affiliations:** 1 Department of Medicine, McMaster University, Hamilton, ON Canada; 2 CHU de Québec Research Centre and Laval University, Québec, QC Canada; 3 Division of Orthopaedic Surgery, Western University, London, ON Canada; 4 Department of Health Research Methods, Evidence and Impact (HEI), McMaster University, Hamilton, ON Canada; 5 Programs for Assessment of Technology in Health, The Research Institute of St. Joe’s Hamilton, St Joseph’s Healthcare Hamilton, Hamilton, ON Canada; 6 Centre for Health Economics and Policy Analysis (CHEPA), McMaster University, Hamilton, ON Canada; 7 Department of Family and Community Medicine, University of Toronto, Toronto, ON Canada; 8 Amgen Inc, California, USA; 9 Amgen Canada Inc., Mississauga, ON Canada

**Keywords:** Osteoporosis, Fragility fracture, Subsequent fracture, Real-world data, Imminent fracture risk, Post fracture care, Secondary fracture prevention

## Abstract

**Background:**

The secondary fracture prevention gap in the osteoporosis field has been previously described as a ‘crisis’. Closing this gap is increasingly important in the context of accumulating evidence showing that an incident fragility fracture is associated with an increased risk of subsequent fracture within 1–2 years, known as imminent fracture risk. The objective of this study was to use health services data to characterize the time between index fragility fractures occurring at different osteoporotic sites and subsequent fractures.

**Methods:**

This retrospective observational study used de-identified health services data from the publicly funded healthcare system in Ontario, the largest province of Canada. Patients aged > 65 with an index fragility fracture occurring between 2011 and 2015 were identified from the ICES Data Repository using International Classification of Diseases (ICD)-10 codes. We examined median time to subsequent fragility fractures for osteoporotic fracture sites until the end of follow-up (2017). BMD assessment and use of osteoporosis therapies following index fracture were also characterized.

**Results:**

Among 115,776 patients with an index fragility fracture, 17.8% incurred a second fragility fracture. Median time between index and second fracture occurring at any site was 555 days (interquartile range: 236–955). For each index fracture site examined, median time from index to second fracture was < 2 years. The proportion of patients with BMD assessment was 10.3% ≤1 year prior to and 16.4% ≤1 year post index fracture. The proportion of patients receiving osteoporosis therapy was 29.8% ≤1 year prior, 34.6% ≤1 year post, and 25.9% > 3 years post index fracture.

**Conclusions:**

This cohort of Canadian patients aged > 65 years who experienced a fragility fracture at any site are at imminent risk of experiencing subsequent fracture within the next 2 years and should be proactively assessed and treated.

**Supplementary Information:**

The online version contains supplementary material available at 10.1186/s12891-021-04051-9.

## Background

Fractures due to osteoporosis have been labelled as a public health crisis in part due to an increasing number of older adults at risk of fragility fractures and low rates of post-fracture management resulting in a large care gap across Europe and North America [1]. In Canada, osteoporosis is responsible for approximately 200,000 cases of fragility fractures annually [2–4] and the incidence of fragility fractures was estimated to be higher than that for myocardial infarction, stroke, and breast cancer combined [2, 3, 5, 6]. The burden of fragility fractures is high in adults aged over 65 years [7, 8] and the predicted increase in the number of older adults (eg, in Canada from 15% in 2011 to 25% in 2031) [9] is expected to result in a proportional increase in the number of fragility fractures in the next decades [10].

An incident fragility fracture is associated with an acute risk of subsequent fracture occurring within 1–2 years, known as an imminent fracture risk [11, 12]. When examining a 10-year period after an incident fracture, it was reported that the majority of subsequent fragility fractures tend to occur within the initial 2 years – 61% of subsequent fractures were reported to occur within the initial 2 years after a hip fracture, 54% after forearm fracture and 53% after humerus fracture (undefined anatomical location) [13]. Studies have shown that a fragility fracture occurring at any site within 1–2 years prior, including non-vertebral sites such as wrist and humerus (proximal or undefined anatomical location), was a better predictor of subsequent fracture risk than a more temporally distant fracture [11, 12, 14–21]. Recent clinical guidelines have therefore recommended urgent initiation of pharmacotherapy in osteoporotic adults who have sustained a fragility fracture at any osteoporotic site in the preceding 2 years [11].

In contrast to osteoporosis clinical practice, diagnosis of an incident event in patients with cardiovascular disease routinely results in urgent initiation of pharmacotherapy to prevent secondary events [22, 23]. In Canada, approximately 90% of patients receive antiplatelet therapy and other secondary prevention measures following acute coronary syndrome to prevent future events [24], whereas only 10–20% of patients receive pharmacotherapy following a fragility fracture [25–27]. Thus, recently published global calls to action have labelled the secondary prevention treatment gap a ‘crisis’ and urged for a shift in focus from managing osteoporosis to managing fragility fractures [28–30].

The objective of this study was to characterize imminent risk using a simple and novel approach, by describing the time to subsequent fracture after index fragility fractures of different sites. Proportions of patients receiving bone mineral density (BMD) assessment and osteoporosis medications pre and post index fracture were also described. A large Canadian patient cohort was drawn from Ontario, a province contributing to more than one-third of osteoporotic fractures occurring in Canada [4] with the aim to provide evidence supporting the urgency of closing the secondary fracture prevention gap as part of the existing calls to action.

## Methods

### Data sources and setting

This was a population-based retrospective database study conducted in Ontario, Canada. Health care encounters were recorded in multiple record-level, administrative datasets in the ICES Data Repository. Encrypted patient-specific identifiers (ICES-specific key number (IKN)) were used to link the administrative datasets [31]. The datasets include health services records for as many as 13 million people living in Ontario [32]. The primary databases used for this study are provided in Supplementary Table 1.

### Study participants

Ontario residents aged > 65 with an index fragility fracture occurring between January 1, 2011 to March 31, 2015 were identified from healthcare records. The cohort was limited to those aged > 65 to collect medication data based on public drug coverage for at least 1 year prior to the index fracture. Data from 5 years prior to the index event, and up to March 31, 2017 were analysed (Supplementary Figure 1). Depending on when the index fracture occurred within the study period, the opportunity for follow up in this cohort ranged between 2 years (2015–2017) and 6 years (2011–2017). Fragility fractures were identified from hospital admissions, emergency and ambulatory care using International Classification of Diseases (ICD)-10 diagnostic codes for fracture as a main diagnosis or admitting diagnosis (see Supplementary Table 2). Patients were excluded from the cohort if they presented with non-osteoporotic fracture sites (ie, skull, face, hands, and feet) or fractures associated with a trauma code to maximize the probability that only fragility fractures were examined (see Supplementary Table 3) [33]. Patients were also excluded if they experienced a fragility fracture within 5 years prior to the index date to minimize the likelihood that examined outcomes were related to a recent fracture occurring prior to an index event.

### Variables of interest and outcome measures

Outcome measures included rate of subsequent fractures and median time between index fractures occurring at different osteoporotic sites (i.e., hip, vertebral [clinical], wrist [distal radius, or both distal radius and ulna], clavicle/ribs/sternum, humerus, tibia/fibula/knee [including medial and lateral malleolus], pelvis, radius/ulna [proximal, midshaft, or distal ulna only], multisite, femur) and subsequent fractures, captured from index fracture until the end of the study period. To avoid double-counting, fracture codes of the same type that occurred within 3 months of each other were assumed to stem from the same fracture. Additional outcome measures included BMD assessment (by dual energy x-ray absorptiometry [DXA] only) and osteoporosis treatments prior to and following the index fracture. The following treatments licensed for use to treat osteoporosis in Canada were included in the analysis: bisphosphonates, denosumab, hormone replacement therapy (HRT), teriparatide and raloxifene. Calcium and vitamin D supplementation was not assessed as these data are not available in the administrative datasets of the ICES Data Repository. DXA-BMD assessment dates were obtained via Ontario Health Insurance Plan (OHIP) billing codes. For subsequent fractures and BMD assessment, all patients had a minimum 1-year of follow-up. Persistence of osteoporosis treatment was assessed and defined as time on any osteoporosis treatment during the study period and based on the number of days supplied with treatment.

### Data synthesis and analysis

The number and proportion of patients with a subsequent fragility fracture and median time from index to subsequent fractures were described in the entire index fracture cohort. The number and proportion of patients with second fragility fractures were also reported in subgroups included in the assessment of BMD and osteoporosis medications. The proportion of patients undergoing BMD screening within 5 years prior and up to 5 years following the index fracture was obtained. The analysis of dispensed osteoporosis treatments included two time periods: (1) prior to and during the time of index event and (2) post index event. The first period included osteoporosis treatments dispensed within 1 year prior to, and at the time of the index event. Post index event dispensed osteoporosis treatments were assessed from 8 days post index fracture hospital discharge date up to 5 years post index fracture. To determine the proportion of patients with subsequent fractures, BMD assessments and osteoporosis treatment, the number of individuals in the specified year was divided by the number of individuals alive at the beginning of that year. Persistence was estimated using a cumulative incidence function adjusted for death as a competing risk; each patient contributed the time of observation from initiation to the end of osteoporosis treatment during the time frame starting from the date of the index fracture until the end of follow up. The REporting of studies Conducted using Observational Routinely-collected health Data (RECORD) Statement was used to report the findings from this study [34].

## Results

### Characteristics of the study cohort

The cohort included 115,776 patients with a fragility fracture (see Supplementary Figure 2). The median age was 81 years (IQR 74–87 years) and 72.3% of patients were women (Table 1). The most common sites of index fractures were the hip (27.3%, *n* = 31,613), wrist (15.4%, *n* = 17,859), and clavicle/ribs/sternum (12.6%, *n* = 14,559). Hip and clinical vertebral fractures vs non-hip non-vertebral fractures vs multisite fractures made up 34.0% (*n* = 39,334) vs 62.8% (*n* = 72,707) vs 3.2% (*n* = 3735) of all index fracture skeletal sites, respectively. The number of index fragility fractures occurring at any site annually ranged from 25,154 in 2011 to 29,385 in 2014. The most common types of comorbidities in this cohort were osteoarthritis (76.2%), diabetes (30.6%), and stroke or cerebrovascular events (30.3%). The proportion of patients on oral steroid treatment 1 year prior to index fracture was 2.9%.

**Table 1:** Characteristics of the index fragility fracture cohort

Characteristic	n (%)^**a**^
**Total number of patients in cohort**	115,776
**Sex**	
Women	83,690 (72.3)
Men	32,086 (27.7)
**Age (years)**	
Median (IQR)	81 (74–87)
Mean (SD)	80.4 (8.3)
66–70	17,998 (15.5)
71–75	17,847 (15.4)
76–80	20,596 (17.8)
81–85	24,119 (20.8)
≥ 86	35,216 (30.4)
**Index fracture by site**^**b**^	
Hip	31,613 (27.3)
Wrist (distal radius, or both distal radius and ulna)	17,859 (15.4)
Clavicle/ribs/sternum	14,559 (12.6)
Humerus	13,237 (11.4)
Tibia/fibula/knee (including medial and lateral malleolus)	10,894 (9.4)
Pelvis	8328 (7.2)
Vertebral (clinical)	7721 (6.7)
Radius/ulna (proximal, midshaft, or distal ulna only)	4828 (4.2)
Multisite	3735 (3.2)
Femur	3002 (2.6)
**Index fracture at any site by year**^**b**^	
2011	25,154
2012	26,045
2013	27,969
2014	29,385
2015^b^	7223
**Index fragility fracture treatment location**	
Urban	103,720 (89.6)
Rural	10,626 (9.2)
Missing	1430 (1.2)
**Respiratory conditions**^**c**^	
Asthma	17,538 (15.1)
COPD	33,485 (28.9)
**Inflammatory conditions**^**c**^	
RA	4459 (3.9)
Psoriasis	8076 (7.0)
SPA	5084 (4.4)
Osteoarthritis	88,223 (76.2)
**Cancer**^**c**^	8390 (7.2)
**Chronic kidney disease**^**c**^	13,757 (11.9)
**Diabetes**^**c**^	35,434 (30.6)
**Vascular events**^**c**^	
MI	8175 (7.1)
Stroke or Cerebrovascular Events	35,030 (30.3)
**Dementia**^**c**^	24,092 (20.8)
**OP treatment**^**d**^	
Any OP treatment	32,757 (28.3)
Bisphosphonate	29,030 (25.1)
Denosumab	1578 (1.4)
HRT	3597 (3.1)
Raloxifene	656 (0.6)
Teriparatide	0 (0)
**Steroid use**^**d**^	3340 (2.9)
**Opioid use**^**d**^	34,834 (30.1)

*Abbreviations*: *COPD* chronic obstructive pulmonary disease, *HRT* hormone replacement therapy, *IQR* interquartile range, *MI* myocardial infarction, *OP* osteoporosis, *RA* rheumatoid arthritis, *SD* standard deviation, *SPA* spondyloarthritis

^a^Values reported as n (%) unless otherwise indicated; percent of total cohort (*N* = 115,776)

^b^Index fragility fracture cases from January 1, 2011 to March 31, 2015

^c^Time frame for cancer was within 5 years prior to index date and for all other comorbidities any time prior to index date

^d^Dispensed within one year prior to index fracture; assessed based on public coverage

### Subsequent fractures

**Fig. 1:**
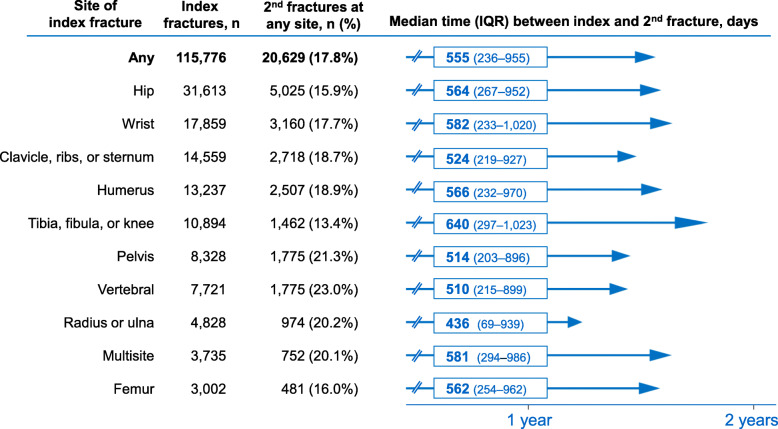
Median time to second fragility fracture occurring at any site (by index fracture site). Number of index fractures, number and proportion of second fragility fractures at any site, and time to second fracture stratified by site of index fracture. Fracture sites are in descending order of number of index fractures. Abbreviations: IQR, interquartile range

Among all patients in the cohort, 17.8% (*n* = 20,629) incurred a second fracture and 3.6% (*n* = 4197) incurred a third fracture over the study period. The median time between index and second fracture of any site was 555 days (interquartile range: 236–955; Fig. 1). When the median time was examined across any index fracture site, it was consistently < 2 years (< 730 days; Fig. 1). Furthermore, the proportion of patients experiencing a second fragility fracture was consistently > 15% across index fracture sites, except for index fractures of the tibia/fibula/knee (13.4%). The most common sites of second fracture were hip (27.8%, *n* = 5745), clavicle/sternum/ribs (11.9%, *n* = 2460), and wrist (10.9%, *n* = 2249), with the combination of hip and clinical vertebral fractures accounting for 36.7% (*n* = 7564) of second fractures (see Supplementary Figure 3).

### Secondary fracture prevention management

**Fig. 2:**
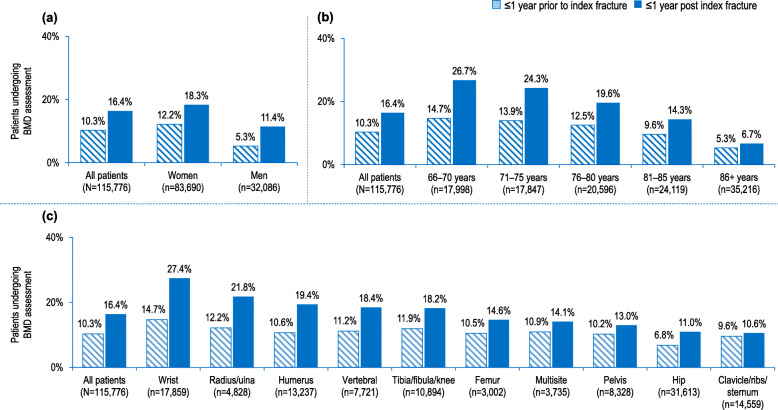
Proportion of fragility fracture patients undergoing BMD assessment ≤1 year prior to and ≤ 1 year post index fracture by: **a** sex; **b** age group; and **c** site of index fracture

The proportion of patients with BMD assessment was 10.3% within 1 year prior and 16.4% within 1 year post index fracture (Fig. 2a). The proportion of patients with BMD assessment post index fracture was the highest at 1-year post facture and decreased with time over 5 years after fracture (from 16.4 to 3.7%) (see Supplementary Figure 4a).

BMD assessment post-fracture was more commonly performed in women (18.3%); 66–70 years age group (26.7%); and for patients with wrist index fracture site (27.4%) (Fig. 2a-c). Among these subgroups, patients in younger age groups or with index wrist fracture had lower absolute risk of second fracture relative to patients in older age groups or those with index fracture occurring at most of the other skeletal sites (Table 2). Further, patients with pelvis or multisite index fractures had among the lowest rates of BMD assessment post-index fracture and among the highest absolute risk of second fracture relative to other index fracture sites, while patients with hip or clavicle/ribs/sternum also had among the lowest BMD rates albeit the risk for subsequent fracture was not among the highest. Finally, men had lower rates of BMD assessment and also lower second fracture risk relative to women.

**Table 2:** Proportion of patients in the index fragility fracture cohort with second fracture occurring at any fracture site, by subgroups

Subgroups (n)^**a**^	Second fracture, any fracture site % (n)^**b**^
**By sex**	
Women (83,690)	19.3% (16,194)
Men (32,086)	13.8% (4435)
**By age category**	
66–70 (17,998)	13.0% (2331)
71–75 (17,847)	15.3% (2737)
76–80 (20,596)	18.3% (3759)
81–85 (24,119)	20.4% (4911)
≥ 86 (35,216)	19.6% (6891)
**By sex and age category**	
Women < 75 years old (*n* = 22,544)	14.7% (3312)
Women ≥75 years old (*n* = 61,146)	21.1% (12,882)
Men < 75 years old (*n* = 9595)	12.0% (1155)
Men ≥75 years old (*n* = 22,491)	14.6% (3280)
**By index fracture site**^**c**^	
Vertebral (7721)	23.0% (1775)
Pelvis (8328)	21.3% (1775)
Radius/ulna (4828)	20.2% (974)
Multisite (3735)	20.1% (752)
Humerus (13,237)	18.9% (2507)
Clavicle/ribs/sternum (14,559)	18.7% (2718)
Wrist (17,859)	17.7% (3160)
Femur (3002)	16.0% (481)
Hip (31,613)	15.9% (5025)
Tibia/fibula/knee (10,894)	13.4% (1462)

^a^ Subgroups included in the assessment of BMD and osteoporosis treatment

^b^ Percent of respective subgroup

^c^ Index fragility fracture cases from January 1, 2011 to March 31, 2015. Second fragility fracture cases from the date of index event to March 31, 2017. Reported from highest to lowest proportion of second fractures

### Osteoporosis therapies

**Fig. 3:**
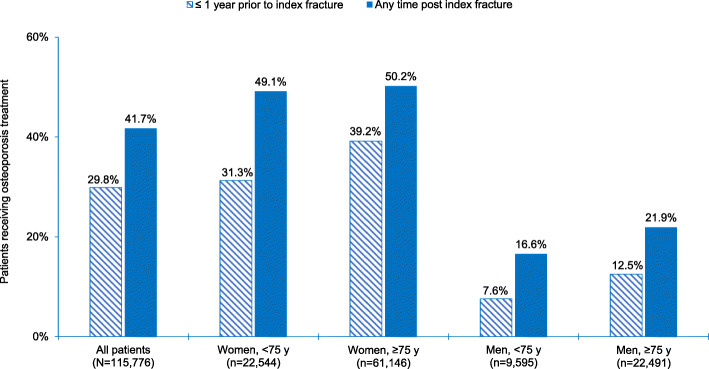
Proportion of patients, by sex and age group, receiving any osteoporosis treatment within 1 year prior* to and during the time of index fragility fracture and at any time post index fracture. * ≤ 1 year prior period included osteoporosis treatments dispensed within 1 year prior to and during the time of index fracture, and also captured the period of 7 days post index fracture hospital discharge date (to reflect a potential delay in the dispensing of osteoporosis treatments prescribed at the time of the index event). Post index event dispensed osteoporosis treatments were assessed from 8 days post index fracture hospital discharge date until the end of study follow-up. Osteoporosis treatments examined in this cohort included bisphosphonates (alendronate, etidronate, risedronate, or zoledronic acid), denosumab, teriparatide, raloxifene, and HRT

The proportion of patients receiving osteoporosis therapy was 29.8% within 1 year prior to and during the time of index fracture, 34.6% within 1 year post, and 25.9% during a period of > 3 years post index fracture. The highest proportion of patients receiving osteoporosis therapy at any time post index fracture (50.2%) or within 1 year post index fracture (42.8%) was among women aged ≥75 (Fig. 3; Supplementary Figure 4b). This subgroup was observed to also have the highest risk of second fracture observed relative to younger women or older/younger men (Table 2). Further, men had 2.3–3.0-fold lower rates of osteoporosis treatment post-index fracture relative to women (Fig. 3), yet their second fracture risk was only 1.2–1.4-fold lower. Of patients who were on osteoporosis therapy at the time of their index fracture, 73.4% (95% confidence interval (CI) 72.8–73.9%) were non-persistent (i.e. ended treatment) over the follow-up period.

## Discussion

In this real-world cohort of patients aged > 65 with an index fragility fracture, 17.8% of patients (*n* = 20,629) sustained a second fracture during the follow-up period. Half of patients who incurred a second fracture, sustained it within 2 years after their index event; this timeframe was consistent across all index fracture sites examined. Hip and clinical vertebral fractures, which were previously reported to increase mortality in Canadian men and women by 2–3 fold [35], were found to make up 34.0% of index and 36.7% of second fractures in this cohort.

Our findings are supported by results of previous studies, where 18% of women ≥65 years and 11–12% of women ≥50 years sustained a second osteoporotic fracture within 2 years of an index fracture [21, 36, 37]. Studies examining fracture risk over time after an incident fracture have demonstrated a 2.7 to 5-fold higher fracture risk within 1–2 years post-fracture (ie, imminent risk) compared to 1.4 to 2-fold higher 10-year risk [12, 38]. The data highlighting high rates of subsequent fractures within 2 years post-fracture, as well as recommendations in recent international guidelines, support the need to recognize a fragility fracture at any site as an important risk factor for subsequent fracture [11, 13, 14, 28, 30].

Our study findings also provide evidence for lack of secondary fracture prevention showing only 16.4% of patients in this cohort undergoing BMD assessment within one-year post-index fracture, which is recommended to help re-assess fracture risk post-fracture [39]. BMD assessment has also been associated with an increased use of osteoporosis therapies and reduction in the rate of hip fractures [40, 41]. Wrist index fracture was the site associated with the highest rates of post-fracture BMD assessment, but was not associated with as high of a risk for subsequent fractures relative to many other index fracture sites. As such, increased awareness among practitioners is needed to recognize fractures at all other osteoporotic sites (except ankle) as a prompt for BMD assessment, as much as, and even more so, than wrist fracture. Although patients in older age groups or with hip index fractures were also associated with lower BMD post-fracture assessment rates relative to their counterparts, the need for post-fracture BMD assessment for the purposes of fracture risk re-assessment is not as high in these groups of patients, because hip fracture alone or older age plus a history of fracture are indicators of high fracture risk independent of BMD [7, 13]. Osteoporosis therapies, which reduce the risk of vertebral and non-vertebral fractures [7, 42, 43] were dispensed ≤1 year prior and during the time of index fracture in 29.8% of patients and in 41.7% of patients any time post index-fracture [24, 44]. Although, based on our data, it was not possible to estimate 10-year FRAX fracture risk for all patients in this cohort, it could be estimated for women aged ≥75. All women aged ≥75 were potentially at high fracture risk in this cohort (based on FRAX calculator inputs of age 75, BMI 25–31 kg/m^2^ and a history of fragility fracture) [45], and should have initiated therapy according to the 2010 Osteoporosis Canada guidelines [7]. However, only 50.2% of women aged ≥75 received therapy over the study follow-up, compared to 39.2% within 1 year prior and during the time of index fracture. Furthermore, recent guidelines recommend urgent initiation of treatment in all adults who have sustained a fragility fracture in the preceding 2 years to help prevent subsequent fractures [11, 13, 43]. Finally, when considering post-fracture treatment gap in relation to subsequent fracture risk, men were observed to have a larger discrepancy between these two outcomes relative to women, and based on these data more awareness is needed among clinical practitioners to recognize a fragility fracture as a disease event requiring appropriate secondary prevention in men.

In our cohort, approximately 73% of patients discontinued osteoporosis treatment by the end of the follow up period. This is higher than rates reported in a recent study of Canadian women and men recruited as part of a Fracture Liaison Service (FLS); 1- and 2-year non-persistence rates were 34 and 46%, respectively [46]. Considering the decline in treatment rates and persistence observed with time after fracture, it is important to note the benefits of long-term osteoporosis treatment highlighted in recent literature [43, 47, 48].

The secondary fracture prevention gap described here may have been influenced by several factors documented in recent studies including: insufficient communication from the fracture clinic informing family doctors of their patient incurring a fragility fracture and of high fracture risk (if present) [49]; not incorporating initiation of osteoporosis treatment into discharge order sets following hip fracture [50]; deprioritization of osteoporosis management over other chronic diseases in primary care potentially due in part to underestimation of the consequences of fragility fractures on morbidity and mortality in elderly people [51]; lack of urgency around secondary fracture prevention by utilizing 10 year fracture risk instead of imminent fracture risk [7]; the overreliance on densitometric osteoporosis diagnosis thresholds (BMD T-score of ≤ − 2.5) for therapy initiation rather than history of fracture [52, 53]; lack of guidance surrounding the benefit of osteoporosis treatment and the risk of rare adverse events from these treatments (ie, atypical femoral fractures and osteonecrosis of the jaw; < 80 per 100,000 person-years) [7, 54]; and overestimated concerns of these rare events by other specialties (i.e., dentists concerned with osteonecrosis of the jaw [54, 55]). Current efforts are urgently needed to help address the secondary prevention care gap and its contributors, as part of new guidelines development, advocacy measures, and other initiatives (eg, FLS) [11, 13, 53, 54, 56].

This study examined patients aged > 65 on the provincial public drug benefit program and close to one-third of patients were aged ≥86, which limited the generalizability of the results to younger Canadians. By excluding patients who had another fracture 5 years prior to their index event, but not beyond those 5 years, the cohort was potentially biased towards an older population. There may be an underestimation of the number of fractures in this cohort, particularly non-hip, considering that only the ‘Most Responsible Diagnosis’ and ‘Pre-Admit Comorbidity’ were used to identify index fractures. Vertebral fractures were likely underestimated considering that two-thirds are typically silent [57, 58]. Medication rates may have been underestimated, since only medications covered through the provincial public drug program were captured. Medications without public coverage during this study period (i.e. denosumab for post-menopausal women with osteoporosis prior to 2012; denosumab for men with osteoporosis) or with restricted reimbursement criteria (i.e. teriparatide) may have been reimbursed through private insurance plans. As in prior healthcare database research, the determination of fragility fracture was based on the exclusion of high-trauma ICD codes and not independent adjudication [33], which may have inaccurately represented the number of fragility fractures in this cohort.

## Conclusion

Patients aged > 65 who have suffered a fragility fracture at any skeletal site are at imminent risk of experiencing subsequent fracture within the next 2 years and should be proactively assessed and treated for osteoporosis [7, 11, 40, 43, 59], considering the large secondary fracture prevention gap highlighted in this retrospective study, in addition to prior research on osteoporosis management in Canada [25–27].

## Supplementary Information

**Additional file 1: Supplementary Table 1.** Primary databases used for the study. **Supplementary Table 2.** Diagnosis Codes for Fragility Fractures. **Supplementary Table 3**. Diagnosis Codes for Trauma Codes. **Supplementary Figure 1.** Study schema. **Supplementary Figure 2.** Flow Diagram of patients included in study. **Supplementary Figure 3.** Number and proportion of patients with second fragility fractures by site of second fracture. **Supplementary Figure 4.** Proportion of patients with (a) BMD assessment, over a period of 5 years prior to and post fracture, by sex and (b) receiving any osteoporosis treatment, by sex and age group, over time*

## Data Availability

The data that support the findings of this study are available from ICES but restrictions apply to the availability of these data, which were used under license for the current study, and so are not publicly available [https://www.ices.on.ca/Data-and-Privacy/ICES-data] [32]. Data are however available from the authors upon reasonable request and with permission of ICES.

## Abbreviations

BMD: bone mineral density
FLS: Fracture Liaison Services
FRAX: Fracture assessment tool
ICD: International classification of diseases
IKN: ICES-specific key number
