# Supplementary material for: Fragility fracture identifies patients at imminent risk for subsequent fracture: real-world retrospective database study in Ontario, Canada
Source: BMC Musculoskelet Disord. 2021 Feb 26;22:224. doi: 10.1186/s12891-021-04051-9 (PMC7908684; doi:10.1186/s12891-021-04051-9)
Supplement: Supplementary file 1 — Additional file 1: Supplementary Table 1. Primary databases used for the study. Supplementary Table 2. Diagnosis Codes for Fragility Fractures. Supplementary Table 3. Diagnosis Codes for Trauma Codes. Supplementary Figure 1. Study schema. Supplementary Figure 2. Flow Diagram of patients included in study. Supplementary Figure 3. Number and proportion of patients with second fragility fractures by site of second fracture. Supplementary Figure 4. Proportion of patients with (a) BMD assessment, over a period of 5 years prior to and post fracture, by sex and (b) receiving any osteoporosis treatment, by sex and age group, over time* [file 12891_2021_4051_MOESM1_ESM.docx]

## Supplementary materials

**Supplementary Table 1:** Primary databases used for the study

**Supplementary Table 2:** Diagnosis Codes for Fragility Fractures

**Supplementary Table 3:** Diagnosis Codes for Trauma Codes

**Supplementary Figure 1:** Study schema

**Supplementary Figure 2:** Flow Diagram of patients included in study

**Supplementary Figure 3:** Number and proportion of patients with second fragility fractures by site of second fracture

**Supplementary Figure 4:** Proportion of patients with (a) BMD assessment, over a period of 5 years prior to and post fracture, by sex and (b) receiving any osteoporosis treatment, by sex and age group, over time*

Supplementary Table 1. Primary databases used for the study

| **Database** | **Type of care/ professions** |
| --- | --- |
| Discharge Abstract Database (DAD)/ Same Day Surgery (SDS) | Inpatient hospital discharge summaries |
| National Ambulatory Care Reporting System (NACRS) | Emergency and ambulatory care visits |
| Ontario Health Insurance Plan (OHIP) | Physician billings to the Ontario Health Insurance Plan (OHIP) |
| Ontario Drug Benefit (ODB) | Prescription drug claims to the Ontario Drug Benefit (ODB) Program for those age 65 and older |

Supplementary Table 2. Diagnosis Codes for Fragility Fractures

| **Fracture type** | **ICD-10 Code** |
| --- | --- |
| Hip | S72.0 - Fracture of neck of femur  S72.1 - Pertrochanteric fracture   - Intertrochanteric fracture - Trochanteric fracture   S72.2 - Subtrochanteric fracture |
| Radius and Ulna | S52.0 - Fracture of upper end of ulna  S52.1 - Fracture of upper end of radius  S52.2 - Fracture of shaft of ulna  S52.3 - Fracture of shaft of radius  S52.4 - Fracture of shafts of both ulna and radius  S52.7 - Multiple fractures of forearm  S52.8 - Fracture of other parts of forearm   - Lower end of ulna - Head of ulna   S52.9 - Fracture of forearm, part unspecified |
| Wrist | S52.5 - Fracture of lower end of radius  S52.6 - Fracture of lower end of both ulna and radius |
| Humerus/ Shoulder | S42.2 - Fracture of upper end of humerus  S42.3 - Fracture of shaft of humerus  S42.4 - Fracture of lower end of humerus  S42.8 - Fracture of other parts of shoulder and upper arm  S42.9 - Fracture of shoulder girdle, part unspecified  Fracture of shoulder NOS |
| Vertebral | S22.0 - Fracture of thoracic vertebra, Fracture of thoracic spine NOS  S22.1 - Multiple fractures of thoracic spine  S32.0 - Fracture of lumbar vertebra, fracture of lumbar spine |
| Femur | S72.3 - Fracture of shaft of femur  S72.4 - Fracture of distal end of femur  S72.7 - Multiple fractures of femur  S72.8 - Fractures of other parts of femur  S72.9 - Fracture of femur, part unspecified   - Applicable To Fracture of thigh NOS Fracture of upper leg NOS - Type 1 exclusion for fracture of hip NOS |
| Other (tibia, fibula, knee) | S82.0 - Fracture of patella   - Kneecap   S82.1 - Fracture of upper end of tibia   - With or without mention of fracture of fibula   S82.2 - Fracture of shaft of tibia   - With or without mention of fracture of fibula   S82.3 - Fracture of lower end of tibia   - With or without mention of fracture of fibula   S82.4 - Fracture of fibula alone  S82.5 - Fracture of medial malleolus   - Tibia involving   S82.6 - Fracture of lateral malleolus   - Fibula involving |
| Sternum, Clavicle and Ribs | S22.2 – Fracture of sternum  S22.3 – Fracture of rib  S22.4 – Multiple fractures of rib  S42.0 - Fracture of clavicle |
| Pelvis | S32.1 - Fracture of sacrum  S32.2 - Fracture of coccyx  S32.3 - Fracture of ilium  S32.4 - Fracture of acetabulum  S32.5 - Fracture of pubis  S32.7 - Multiple fractures of lumbar spine and pelvis  S32.8 - Fracture of other and unspecified parts of lumbar spine and pelvis   - Fracture of: - ischium - lumbosacral spine NOS - pelvis NOS |
| Multiple Fracture | S42.7 - Multiple fractures of clavicle, scapula and humerus  T02.1 - Fractures involving thorax with lower back and pelvis  T02.2 - Fractures involving multiple regions of one upper limb  T02.3 - Fractures involving multiple regions of one lower limb  T02.4 - Fractures involving multiple regions of both upper limbs  T02.5 - Fractures involving multiple regions of both lower limbs  T02.6 - Fractures involving multiple regions of upper limb(s) with lower limb(s)  T02.7 - Fractures involving thorax with lower back and pelvis with limb(s)  T02.8 - Fractures involving other combinations of body regions  T02.9  - Multiple fractures, unspecified |

Supplementary Table 3. Diagnosis Codes for Trauma Codes

| **Trauma Code** | **ICD-10 Code** |
| --- | --- |
| Accidents | V01-V99, X00-X58, X59.9, W20-W99 |
| Injuries involving multiple body regions | T00-T01, T03-T07 |
| Falls | W02-W04, W09, W11-W17 |

Supplementary Figure 1. Study Schema

Index event identification period

(Jan 1, 2011 to Mar 31, 2015)

5-Year lookback period

(Jan 1, 2006 to Jan 1, 2011)

Data collection period

Maximum follow-up date

(Mar 31, 2017)

INDEX EVENT DATE

Note: Index event definition include fracture occurring at a fragility fracture site, excluding non-osteoporotic fracture sites (ie, skull, face, hands, and feet) or fractures associated with a trauma code

Exclusions: invalid linkage number (33); event date after the date of death; missing age or sex; older than 105 years; non-resident of Ontario

Supplementary Figure 2. Flow diagram of patients included in the study

Included in assessment
(n=115,776)

Excluded (n=345,828)

Aged < 66 years (n=310,338)

Death date prior to index fracture (n=78)

Non-Ontario resident (n=123)

Fracture associated with a trauma code (n=21,113)

Fragility fracture within 5 years prior to index date (n=14,176)

Assessed for eligibility^a^
(N=461,604)

^a^All patients with valid IKN with all patients with valid IKN with a fracture occurring at an osteoporotic fracture site (hip, humerus, vertebral, wrist, pelvis, femur, clavicle, ribs/sternum, radius/ulna, or tibia/ fibula/knee) between January 1, 2011 and March 31, 2015. Fractures were identified using ICD-10-CA codes from hospital admissions, emergency room visits, and ambulatory care.

**Abbreviations**: ICD-10-CA, International Classification of Diseases, 10th revision, Canada; IKN, Institute for Clinical Evaluative Sciences (ICES) key number.

Supplementary Figure 3. Number and proportion of patients with second fragility fractures by site of second fracture

Site of second fractures


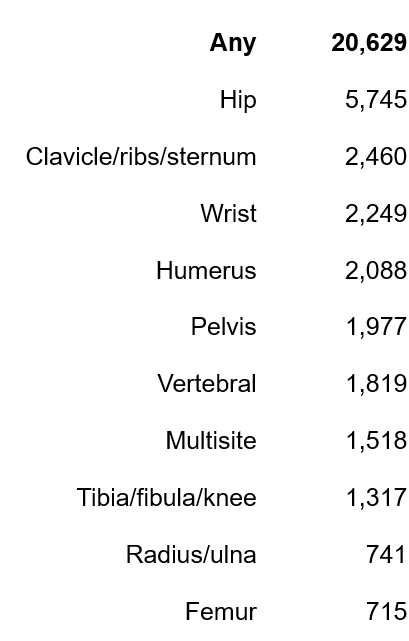


Second fractures, n (%)

Supplementary Figure 4. Proportion of patients with (a) BMD assessment, over a period of 5 years prior to and post fracture, by sex and (b) receiving any osteoporosis treatment, by sex and age group, over time*


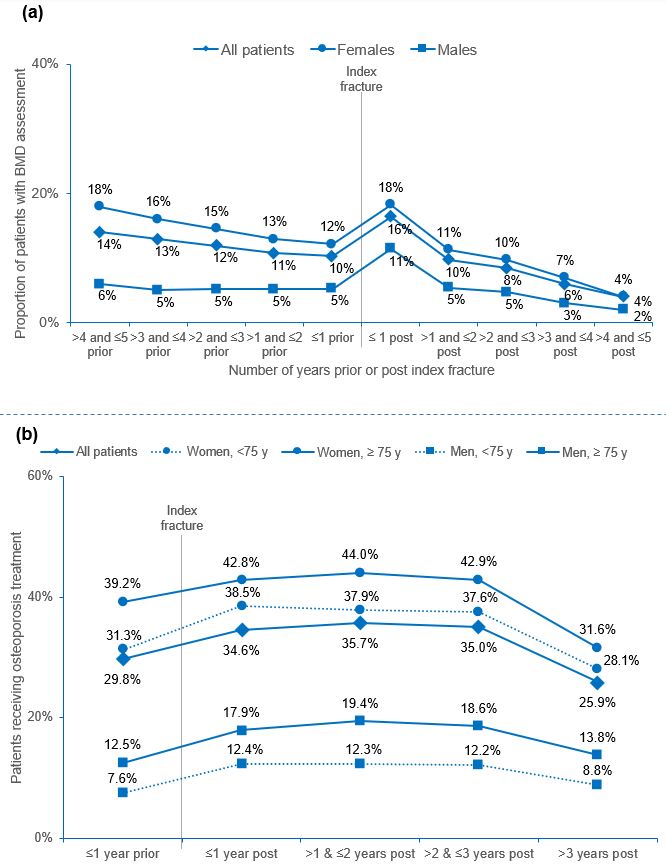


*≤1 year prior period included osteoporosis treatments dispensed within 1 year prior to and during the time of index fracture, and also captured the period of 7 days post index fracture hospital discharge date (to reflect a potential delay in the dispensing of osteoporosis treatments prescribed at the time of the index event). Post index event dispensed osteoporosis treatments were assessed from 8 days post index fracture hospital discharge date until 1 year post index fracture (ie, ≤1 year post), as well as and up to 5 years post index fracture. Osteoporosis treatments examined in this cohort included bisphosphonates (alendronate, etidronate, risedronate, or zoledronic acid), denosumab, teriparatide, raloxifene, and HRT.
